# Supplementary material for: Flavonoid intake is associated with lower mortality in the Danish Diet Cancer and Health Cohort
Source: Nat Commun. 2019 Aug 13;10:3651. doi: 10.1038/s41467-019-11622-x (PMC6692395; doi:10.1038/s41467-019-11622-x)
Supplement: Supplementary file 1 — Supplementary Information [file 41467_2019_11622_MOESM1_ESM.pdf]

Flavonoid intake is associated with lower mortality in the Danish Diet Cancer and Health Cohort

Bondonno et al.

Supplementary Table 1. Pearson's correlations between flavonoid subclasses

	Total flavonoids	Flavonols	Flavanones	Anthocyanidins	Flavones	Flavanol monomers
Total flavonoids	1.00					
Flavonols	0.94*	1.00				
Flavanones	0.15*	0.05*	1.00			
Anthocyanidins	0.25*	0.12*	0.06*	1.00		
Flavones	0.23*	0.13*	0.76*	0.04*	1.00	
Flavanol monomers	0.91*	0.97*	0.01	0.04*	0.08*	1.00
Flavanol oligo+polymers	0.90*	0.74*	0.09*	0.29*	0.21*	0.66*

*$p<0.05$

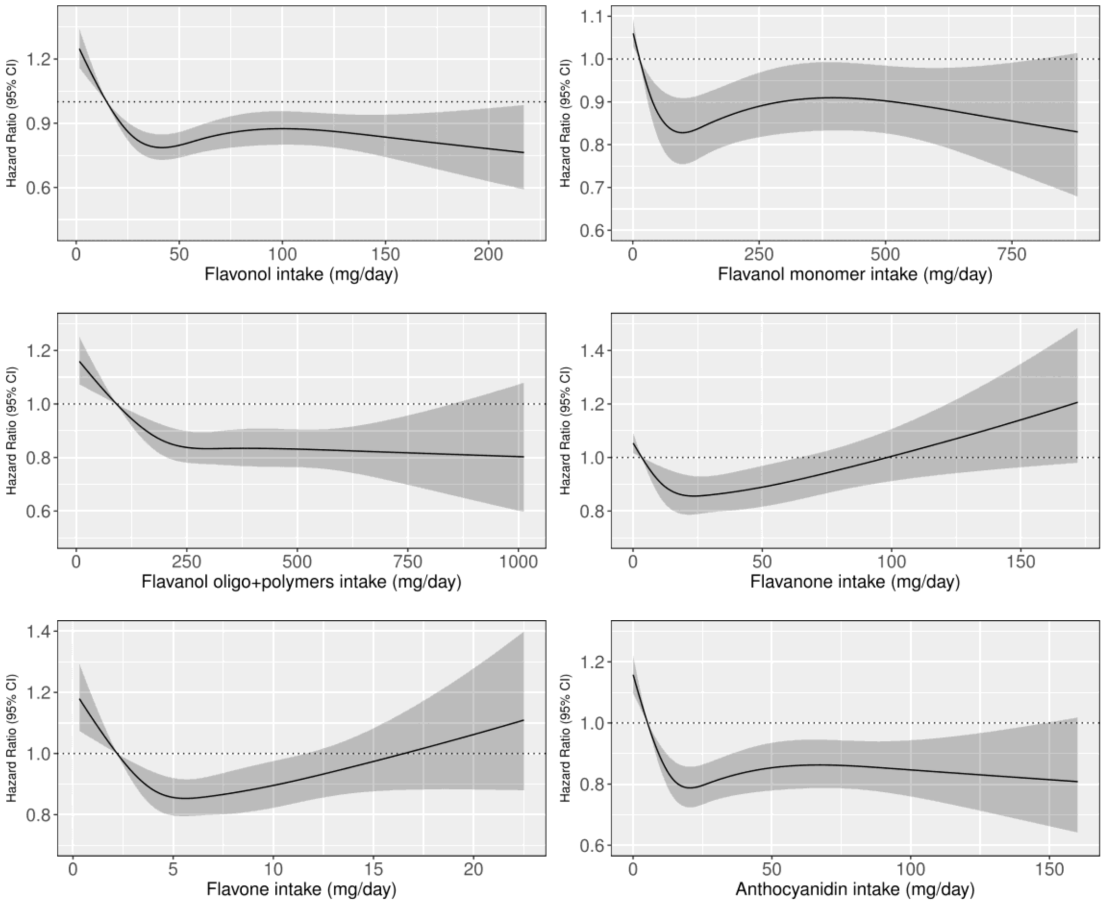

Supplementary Figure 1. Hazard ratios based on cubic spline curves to describe the association between flavonoid subclass intakes (mg/day) and cardiovascular disease-related mortality (n=4 065) among participants of the Danish Diet, Cancer and Health cohort without cardiovascular disease at baseline (n=52 492). Hazard ratios are based on Cox proportional hazards models adjusted for age, sex, BMI, smoking status, physical activity, alcohol intake, hypertension, hypercholesterolemia, social economic status (income) and prevalent disease and are comparing the specific level of flavonoid intake (horizontal axis) to the median intake for participants in the lowest intake quintile.

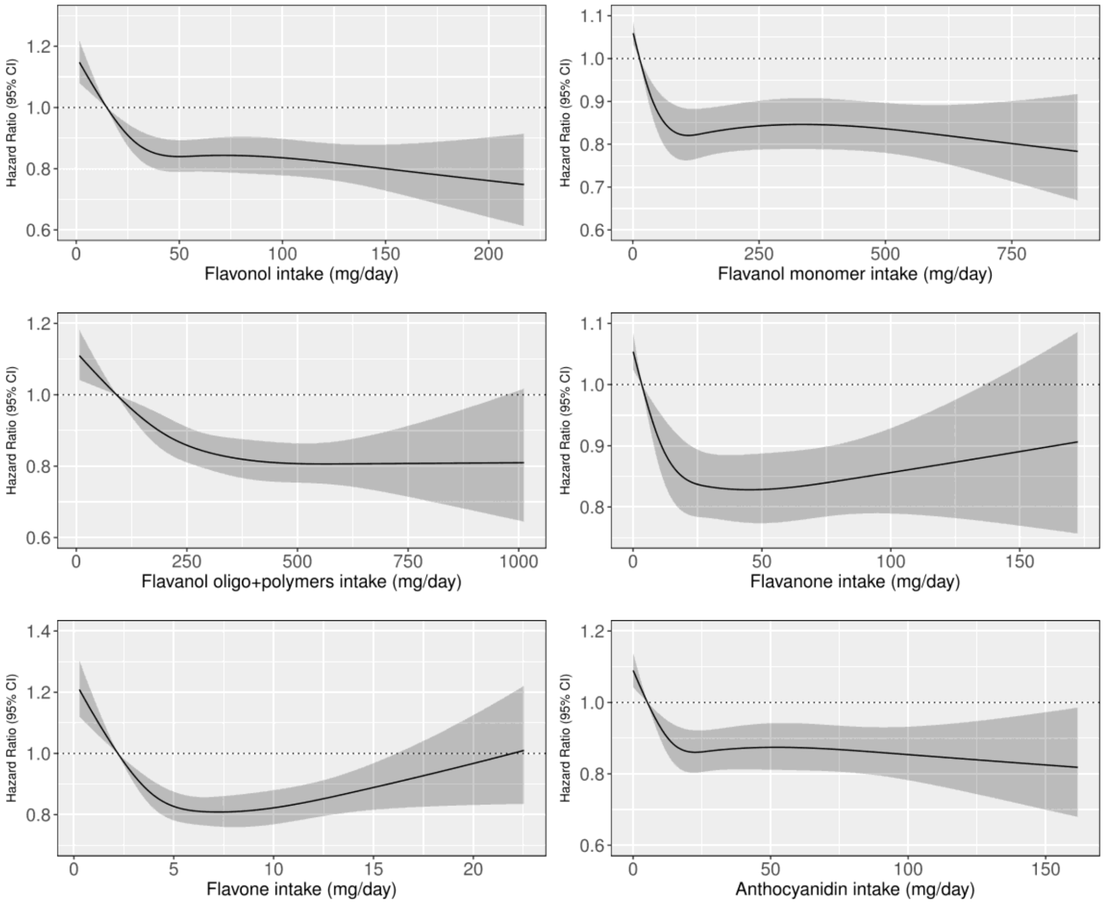

Supplementary Figure 2. Hazard ratios based on cubic spline curves to describe the association between flavonoid subclass intakes (mg/day) and cancer-related mortality (n=6 299) among participants of the Danish Diet, Cancer and Health cohort without cancer at baseline (n=55 801). Hazard ratios are based on Cox proportional hazards models adjusted for age, sex, BMI, smoking status, physical activity, alcohol intake, hypertension, hypercholesterolemia, social economic status (income) and prevalent disease and are comparing the specific level of flavonoid intake (horizontal axis) to the median intake for participants in the lowest intake quintile.

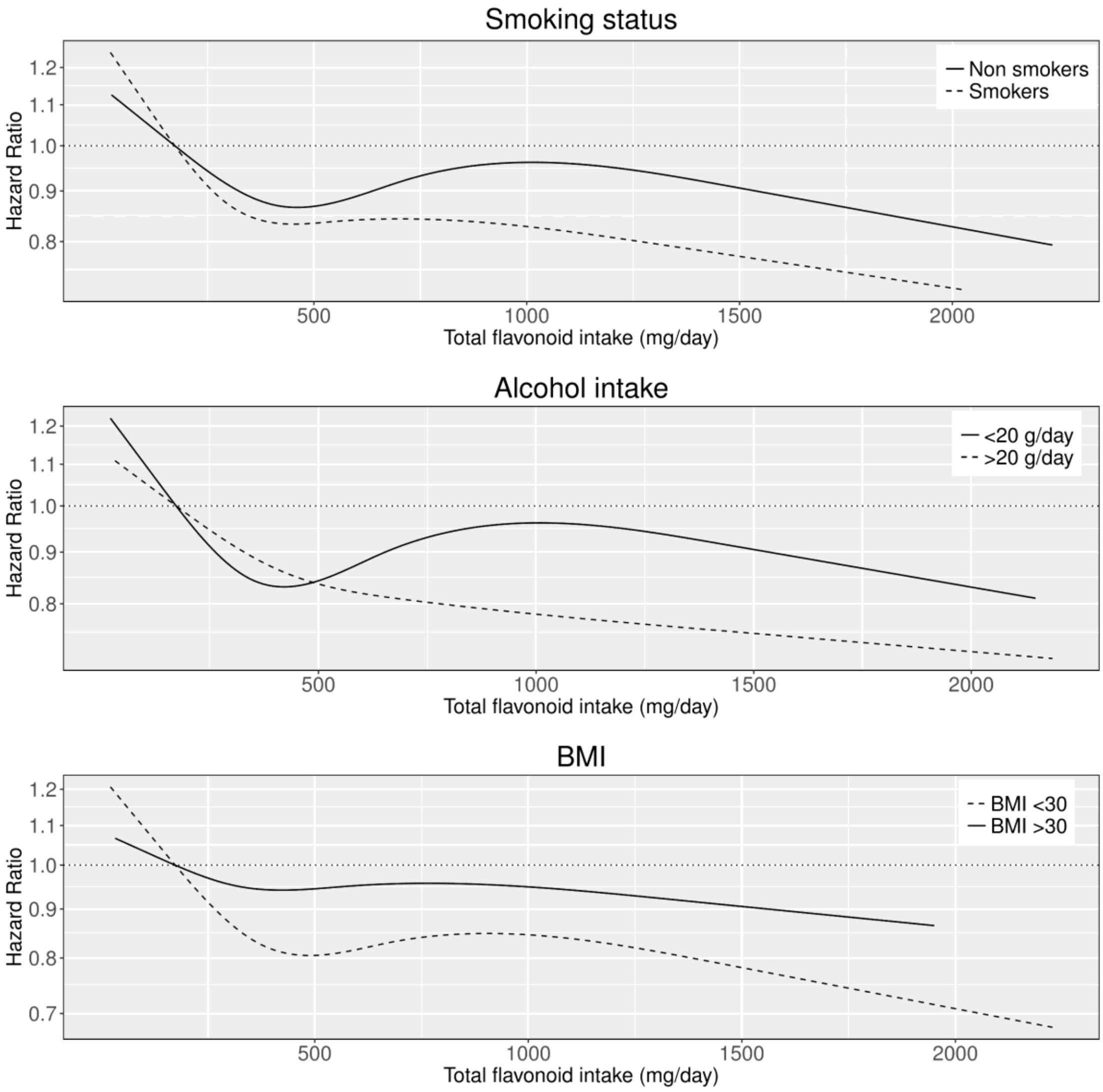

Supplementary Figure 3. Multivariable-adjusted association between total flavonoid intake and cardiovascular disease-related mortality stratified by current smoking status, alcohol intake and BMI among participants without cardiovascular disease at baseline. Values are hazards ratios and 95% CI for the highest compared to the lowest quintiles of intake. All analyses were standardized for age, sex, BMI, physical activity, alcohol intake, hypertension, hypercholesterolemia, smoking status, social economic status (income) and prevalent disease, not including the stratification variable for the subgroups.

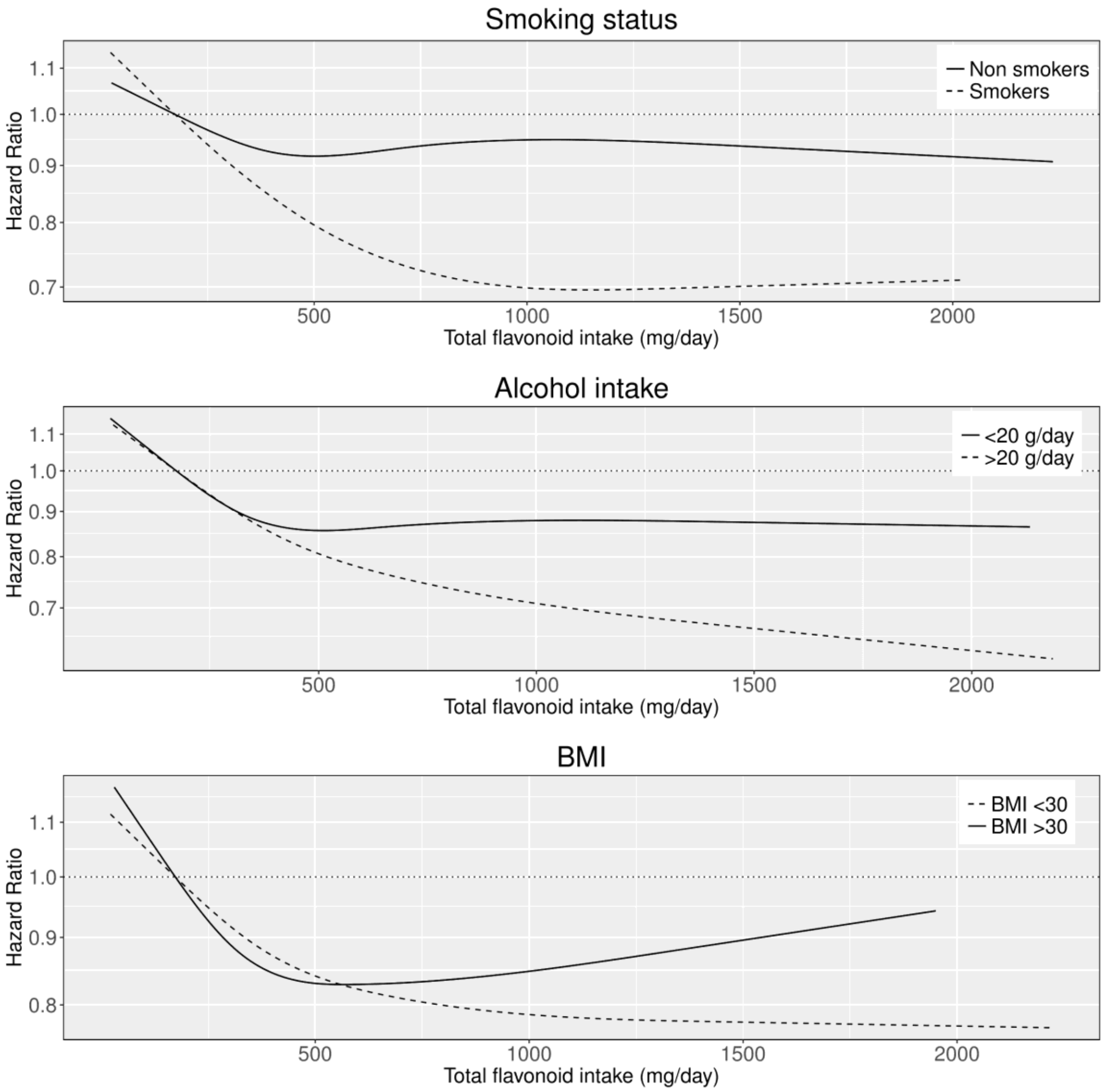

Supplementary Figure 4. Multivariable-adjusted association between total flavonoid intake and cancer-related mortality stratified by current smoking status, alcohol intake and BMI among participants without cancer at baseline. Values are hazards ratios and 95% CI for the highest compared to the lowest quintiles of intake. All analyses were standardized for age, sex, BMI, physical activity, alcohol intake, hypertension, hypercholesterolemia, smoking status, social economic status (income) and prevalent disease, not including the stratification variable for the subgroups.

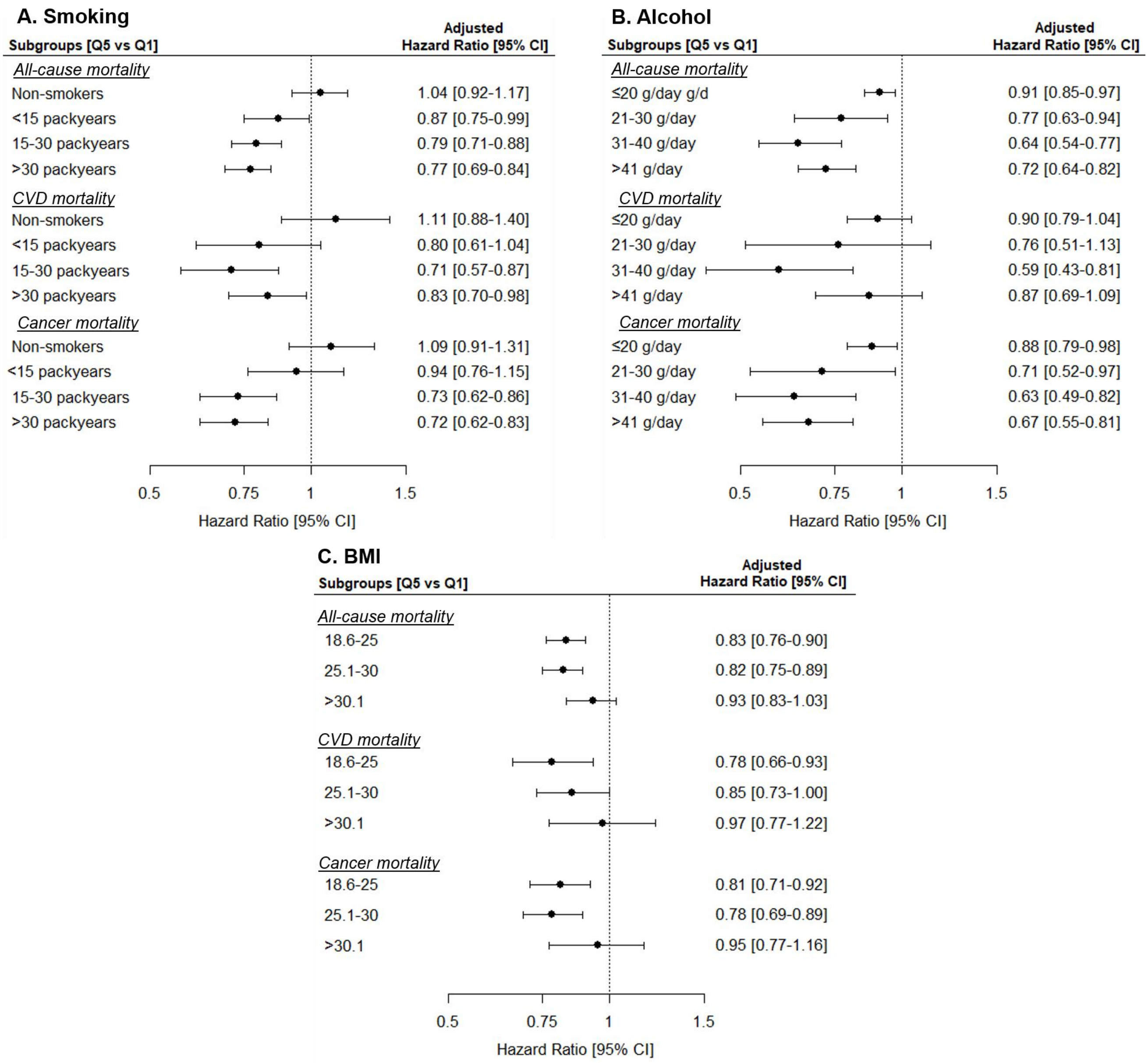

Supplementary Figure 5. Multivariable-adjusted association between total flavonoid intake and all-cause, cardiovascular disease-related, and cancer-related mortality stratified by current smoking intensity (**A**), alcohol intake (**B**) and BMI (**C**). Values are hazards ratios and 95% CI for the highest compared to the lowest quintiles of intake. All analyses were standardized for age, sex, BMI, physical activity, alcohol intake, hypertension, hypercholesterolemia, smoking status, social economic status (income) and prevalent disease, not including the stratification variable for the subgroups.

Supplementary Table 2. Hazard ratios of all-cause mortality by quintiles of total flavonoid intake using alternative models of adjustment

	Total flavonoid intake quintiles				
	**Q1**	**Q2**	**Q3**	**Q4**	**Q5**
Model 2	ref.	**0.88 (0.85, 0.91)**	**0.83 (0.80, 0.86)**	**0.83 (0.80, 0.87)**	**0.83 (0.80, 0.87)**
Model 2b	ref.	**0.88 (0.85, 0.91)**	**0.83 (0.80, 0.86)**	**0.83 (0.80, 0.87)**	**0.83 (0.79, 0.87)**
Model 2c	ref.	**0.88 (0.85, 0.90)**	**0.82 (0.79, 0.85)**	**0.82 (0.79, 0.86)**	**0.83 (0.79, 0.87)**
Model 2d	ref.	**0.88 (0.86, 0.91)**	**0.83 (0.80, 0.86)**	**0.83 (0.80, 0.87)**	**0.83 (0.80, 0.87)**
Model 2*	ref.	**0.88 (0.85, 0.91)**	**0.82 (0.79, 0.86)**	**0.82 (0.78, 0.86)**	**0.82 (0.78, 0.86)**

Hazard ratios (95% CI) for 23-year all-cause mortality obtained from restricted cubic splines based on Cox proportional hazards models. Model 2 (original model) adjusted for age, sex, BMI, smoking status, physical activity, alcohol intake, hypertension, hypercholesterolemia, social economic status (income), diabetes and prevalent disease; Model 2b: Model 2 plus energy intake; Model 2c: Model 2 without adjustment for prevalent diseases which may potentially lie on the causal pathway (adjusted for age, sex, BMI, smoking status, physical activity, alcohol intake, and, social economic status (income) only); Model 2d: Model 2 plus medication use (statins, antihypertensive medication, insulin and aspirin). *Analysis run excluding n=5 492 participants with comorbidities at baseline.

Supplementary Table 3. Hazard ratios of all-cause mortality by quintiles of total flavonoid intake stratified by fruit and vegetable consumption

**Total fruit and vegetable intake tertiles**	Total flavonoid intake quintiles				
	**Q1**	**Q2**	**Q3**	**Q4**	**Q5**
1	ref.	**0.93 (0.86, 0.99)**	**0.89 (0.82, 0.96)**	**0.87 (0.79, 0.96)**	**0.88 (0.80, 0.97)**
2	ref.	**0.84 (0.77, 0.92)**	**0.87 (0.79, 0.96)**	**0.81 (0.73, 0.89)**	**0.79 (0.71, 0.87)**
3	ref.	0.85 (0.72, 1.00)	0.86 (0.73, 1.00)	0.85 (0.73, 1.00)	0.91 (0.78, 1.06)

Hazard ratios (95% CI) for 23-year all-cause mortality obtained from restricted cubic splines based on Cox proportional hazards models and stratified by tertiles of total fruit and vegetable intake. Analyses are adjusted for age, sex, BMI, smoking status, physical activity, alcohol intake, hypertension, hypercholesterolemia, social economic status (income), diabetes and prevalent disease.

Supplementary Table 4. Hazard ratios of burns and foreign objects by quintiles of total flavonoid intake

	Total flavonoid intake quintiles				
	**Q1**	**Q2**	**Q3**	**Q4**	**Q5**
Model 2	ref.	0.95 (0.89, 1.02)	0.94 (0.86, 1.03)	0.97 (0.88, 1.07)	1.01 (0.92, 1.12)

Hazard ratios (95% CI) for any emergency, inpatient, or outpatient visit for a burn or foreign object (n=3 020) during 23 years of follow-up, obtained from restricted cubic splines based on Cox proportional hazards models. Analyses are adjusted for age, sex, BMI, smoking status, physical activity, alcohol intake, hypertension, hypercholesterolemia, social economic status (income), diabetes and prevalent disease.

Supplementary Table 5. Flavonoid intake in study population

Flavonoid class	Constituent compounds
Flavonols	6,8-Dihydroxykaempferol, Kaempferol, Kaempferol 3,7,4-O-triglucoside, Kaempferol 3,7-O-diglucoside, Kaempferol 3-O-(6-malonyl-glucoside), Kaempferol 3-O-acetyl-glucoside, Kaempferol 3-O-galactoside, Kaempferol 3-O-glucoside, Kaempferol 3-O-glucosyl-rhamnosyl-galactoside, Kaempferol 3-O-glucosyl-rhamnosyl-glucoside, Kaempferol 3-O-glucuronide, Kaempferol 3-O-rhamnoside, Kaempferol 3-O-rhamnosyl-rhamnosyl-glucoside, Kaempferol 3-O-rutinoside, Kaempferol 3-O-sophoroside, Kaempferol 3-O-sophoroside 7-O-glucoside, Kaempferol 3-O-xylosyl-glucoside, Kaempferol 3-O-xylosyl-rutinoside, Morin, Myricetin, Myricetin 3-O-arabinoside, Myricetin 3-O-glucoside, Myricetin 3-O-rhamnoside, Myricetin 3-O-rutinoside, Quercetin, Quercetin 3,4-O-diglucoside, Quercetin 3-O-(6-malonyl-glucoside), Quercetin 3-O-(6-malonyl-glucoside) 7-O-glucoside, Quercetin 3-O-acetyl-rhamnoside, Quercetin 3-O-arabinoside, Quercetin 3-O-galactoside, Quercetin 3-O-glucoside, Quercetin 3-O-glucosyl-rhamnosyl-galactoside, Quercetin 3-O-glucosyl-rhamnosyl-glucoside, Quercetin 3-O-glucosyl-xyloside, Quercetin 3-O-glucuronide, Quercetin 3-O-rhamnoside, Quercetin 3-O-rhamnosyl-galactoside, Quercetin 3-O-rutinoside, Quercetin 3-O-sophoroside, Quercetin 3-O-xyloside, Quercetin 3-O-xylosyl-glucuronide, Quercetin 3-O-xylosyl-rutinoside, Quercetin 4-O-glucoside, 3,7-Dimethylquercetin, 3-Methoxynobiletin, 3-Methoxysinensetin, 5,3,4-Trihydroxy-3-methoxy-6:7-methylenedioxyflavone 4-O-glucuronide, 5,4-Dihydroxy-3,3-dimethoxy-6:7-methylenedioxyflavone 4-O-glucuronide, Isorhamnetin, Isorhamnetin 3-O-galactoside, Isorhamnetin 3-O-glucoside, Isorhamnetin 4-O-glucoside, Jaceidin 4-O-glucuronide, Patuletin 3-O-(2-feruloylglucosyl)(1->6)-[apiosyl(1->2)]-glucoside, Patuletin 3-O-glucosyl-(1->6)-[apiosyl(1->2)]-glucoside, Spinacetin 3-O-(2-feruloylglucosyl)(1->6)-[apiosyl(1->2)]-glucoside, Spinacetin 3-O-(2-p-coumaroylglucosyl)(1->6)-[apiosyl(1->2)]-glucoside, Spinacetin 3-O-glucosyl-(1->6)-[apiosyl(1->2)]-glucoside, Spinacetin 3-O-glucosyl-(1->6)-glucoside.
Flavan-3-ols	
Flavanol monomers	(-)-Epicatechin, (-)-Epicatechin 3-O-gallate, (-)-Epigallocatechin, (-)-Epigallocatechin 3-O-gallate, (+)-Catechin, (+)-Catechin 3-O-gallate, (+)-Catechin 3-O-glucose, (+)-Gallocatechin, (+)-Gallocatechin 3-O-gallate, (-)-Epicatechin-(2a-7)(4a-8)-epicatechin 3-O-galactoside.
Flavanol oligo+polymers	Procyanidin dimer B1, Procyanidin dimer B2, Procyanidin dimer B3, Procyanidin dimer B4, Procyanidin dimer B5, Procyanidin dimer B7, Prodelphinidin dimer B3, Procyanidin trimer C1, Procyanidin trimer C2, Procyanidin trimer EEC, Procyanidin trimer T2, Prodelphinidin trimer C-GC-C, Prodelphinidin trimer GC-C-C, Prodelphinidin trimer GC-GC-C, Cinnamtannin A2, 02 mers, 03 mers, 04-06 mers, 07-10 mers, Polymers (>10 mers), Theaflavin, Theaflavin 3,3-O-digallate, Theaflavin 3-O-gallate.

Flavones	Apigenin, Apigenin 6,8-C-arabinoside-C-glucoside, Apigenin 6,8-C-galactoside-C-arabinoside, Apigenin 6,8-di-C-glucoside, Apigenin 6-C-glucoside, Apigenin 7-O-apiosyl-glucoside, Apigenin 7-O-glucoside, Apigenin 7-O-glucuronide, Baicalein, Chrysin, Isorhoifolin, Luteolin, Luteolin 6-C-glucoside, Luteolin 7-O-(2-apiosyl-6-malonyl)-glucoside, Luteolin 7-O-diglucuronide, Luteolin 7-O-glucoside, Luteolin 7-O-glucuronide, Luteolin 7-O-rutinoside, Rhoifolin, Cirsimaritin, Diosmin, Hispidulin, Neodiosmin, Nobiletin, Sinensetin, Tangeretin, Tetramethylscutellarein.
Flavanones	Didymin, Eriocitrin, Eriodictyol, Hesperidin, Naringenin, Naringenin 7-O-glucoside, Naringin, Narirutin, Neoeriocitrin, Neohesperidin, Poncirin, 6-Geranylnaringenin, 6-Prenylnaringenin, 8-Prenylnaringenin, Isoxanthohumol, Hesperetin.
Isoflavones	6-O-Acetyldaidzin, 6-O-Acetylgenistin, 6-O-Malonyldaidzin, 6-O-Malonylgenistin, Daidzein, Daidzin, Genistein, Genistin, 6-O-Acetylglycitin, 6-O-Malonylglycitin, Biochanin A, Glycitein, Glycitin.
Anthocyanins	Cyanidin, Cyanidin 3,5-O-diglucoside, Cyanidin 3-O-(6-acetyl-galactoside), Cyanidin 3-O-(6-acetyl-glucoside), Cyanidin 3-O-(6-caffeoyl-glucoside), Cyanidin 3-O-(6-dioxalyl-glucoside), Cyanidin 3-O-(6-malonyl-glucoside), Cyanidin 3-O-(6-p-coumaroyl-glucoside), Cyanidin 3-O-(6-succinyl-glucoside), Cyanidin 3-O-arabinoside, Cyanidin 3-O-galactoside, Cyanidin 3-O-glucoside, Cyanidin 3-O-glucosyl-rutinoside, Cyanidin 3-O-rutinoside, Cyanidin 3-O-sambubioside, Cyanidin 3-O-sambubiosyl 5-O-glucoside, Cyanidin 3-O-sophoroside, Cyanidin 3-O-xyloside, Cyanidin 3-O-xylosyl-rutinoside, Delphinidin 3-O-(6-acetyl-galactoside), Delphinidin 3-O-(6-acetyl-glucoside), Delphinidin 3-O-(6-p-coumaroyl-glucoside), Delphinidin 3-O-arabinoside, Delphinidin 3-O-feruloyl-glucoside, Delphinidin 3-O-galactoside, Delphinidin 3-O-glucoside, Delphinidin 3-O-rutinoside, Delphinidin 3-O-sambubioside, Malvidin 3,5-O-diglucoside, Malvidin 3-O-(6-acetyl-galactoside), Malvidin 3-O-(6-acetyl-glucoside), Malvidin 3-O-(6-caffeoyl-glucoside), Malvidin 3-O-(6-p-coumaroyl-glucoside), Malvidin 3-O-arabinoside, Malvidin 3-O-galactoside, Malvidin 3-O-glucoside, Pelargonidin, Pelargonidin 3,5-O-diglucoside, Pelargonidin 3-O-(6-malonyl-glucoside), Pelargonidin 3-O-(6-succinyl-glucoside), Pelargonidin 3-O-arabinoside, Pelargonidin 3-O-glucoside, Pelargonidin 3-O-glucosyl-rutinoside, Pelargonidin 3-O-rutinoside, Pelargonidin 3-O-sophoroside, Peonidin, Peonidin 3-O-(6-acetyl-galactoside), Peonidin 3-O-(6-acetyl-glucoside), Peonidin 3-O-(6-p-coumaroyl-glucoside), Peonidin 3-O-arabinoside, Peonidin 3-O-galactoside, Peonidin 3-O-glucoside, Peonidin 3-O-rutinoside, Petunidin 3-O-(6-acetyl-galactoside), Petunidin 3-O-(6-acetyl-glucoside), Petunidin 3-O-(6-p-coumaroyl-glucoside), Petunidin 3-O-arabinoside, Petunidin 3-O-galactoside, Petunidin 3-O-glucoside, Petunidin 3-O-rutinoside, Pigment A, Pinotin A, Vitisin A.
Dihydrochalcones	3-Hydroxyphloretin 2-O-glucoside, Phloretin 2-O-xylosyl-glucoside, Phloridzin.
Dihydroflavonols	Dihydromyricetin 3-O-rhamnoside, Dihydroquercetin 3-O-rhamnoside.
Chalcones	Xanthohumol

Supplementary Table 6. International Classification of Disease codes used to determine prevalent disease, cause specific mortality, and falsification endpoint

**Disease and cause specific mortality**	**ICD codes (ICD-8; ICD-10)**
Ischemic heart disease	410-414; I20-I25
Ischemic stroke	433-434; I63
Peripheral artery disease	440-444; I70-I74
Heart failure	4270-4271; I42, I50, I110, J81
Atrial fibrillation	42793-42794; I48
Chronic kidney disease	580-584; N02-N08, N11-N12, N14, N18-N19, N26, N158-N160, N162-N164, N168, Q61, E102, E112, E132, E142, I120, M321B
Chronic obstructive pulmonary disease	491-493; J42-J44
Cancers	140-209; C00-C99
Cardiovascular disease related mortality	I00-I99
Cancer-related mortality	C00-C99
Burns	DT2, DT3
Foreign bodies	DT15-DT19
