# Supplementary material for: Flavonoid intake is associated with lower mortality in the Danish Diet Cancer and Health Cohort
Source: Nat Commun. 2019 Aug 13;10:3651. doi: 10.1038/s41467-019-11622-x (PMC6692395; doi:10.1038/s41467-019-11622-x)
Supplement: Supplementary file 3 — Reporting Summary [file 41467_2019_11622_MOESM3_ESM.pdf]

nature research

Corresponding author(s): Nicola Bondonno

Last updated by author(s): Jun 19, 2019

Reporting Summary

Nature Research wishes to improve the reproducibility of the work that we publish. This form provides structure for consistency and transparency in reporting. For further information on Nature Research policies, see Authors & Referees and the Editorial Policy Checklist.

Statistics

For all statistical analyses, confirm that the following items are present in the figure legend, table legend, main text, or Methods section.

n/a	Confirmed	
☐	☒	The exact sample size (*n*) for each experimental group/condition, given as a discrete number and unit of measurement
☐	☒	A statement on whether measurements were taken from distinct samples or whether the same sample was measured repeatedly
☐	☒	The statistical test(s) used AND whether they are one- or two-sided<br>*Only common tests should be described solely by name; describe more complex techniques in the Methods section.*
☐	☒	A description of all covariates tested
☐	☒	A description of any assumptions or corrections, such as tests of normality and adjustment for multiple comparisons
☐	☒	A full description of the statistical parameters including central tendency (e.g. means) or other basic estimates (e.g. regression coefficient) AND variation (e.g. standard deviation) or associated estimates of uncertainty (e.g. confidence intervals)
☐	☒	For null hypothesis testing, the test statistic (e.g. *F*, *t*, *r*) with confidence intervals, effect sizes, degrees of freedom and *P* value noted<br>*Give P values as exact values whenever suitable.*
☒	☐	For Bayesian analysis, information on the choice of priors and Markov chain Monte Carlo settings
☒	☐	For hierarchical and complex designs, identification of the appropriate level for tests and full reporting of outcomes
☐	☒	Estimates of effect sizes (e.g. Cohen's *d*, Pearson's *r*), indicating how they were calculated

Our web collection on statistics for biologists contains articles on many of the points above.

Software and code

Policy information about availability of computer code

Data collection	Any request on data collection should be addressed to Danish Cancer Society https://www.cancer.dk/international/ and Statistics Denmark https://www.dst.dk/en.aspx#.<br>Due to restrictions related to Danish law and protecting patient privacy, the combined set of data as used in this study can only be made available through a trusted third party, Statistics Denmark. This state organisation holds the data used for this study.
Data analysis	Software used was: Team RC. R: A Language and Environment for Statistical Computing. 2019.<br>The computer code used in this study can only be made available through a trusted third party, Statistics Denmark https://www.dst.dk/en.aspx#. This state organisation holds the computer codes used for this study.

For manuscripts utilizing custom algorithms or software that are central to the research but not yet described in published literature, software must be made available to editors/reviewers. We strongly encourage code deposition in a community repository (e.g. GitHub). See the Nature Research guidelines for submitting code & software for further information.

Data

Policy information about availability of data

All manuscripts must include a data availability statement. This statement should provide the following information, where applicable:

- Accession codes, unique identifiers, or web links for publicly available datasets
- A list of figures that have associated raw data
- A description of any restrictions on data availability

Due to restrictions related to Danish law and protecting patient privacy, the combined set of data as used in this study can only be made available through a trusted third party, Statistics Denmark. This state organisation holds the data used for this study. University-based Danish scientific organisations can be authorized to work with data within Statistics Denmark and such organisation can provide access to individual scientists inside and outside of Denmark. Requests for data may be sent to Statistics Denmark: https://www.dst.dk/en/kontakt or the Danish Data Protection Agency: https://www.datatilsynet.dk/english.

Field-specific reporting

Please select the one below that is the best fit for your research. If you are not sure, read the appropriate sections before making your selection.

☐ Life sciences ☒ Behavioural & social sciences ☐ Ecological, evolutionary & environmental sciences

For a reference copy of the document with all sections, see nature.com/documents/nr-reporting-summary-flat.pdf

Behavioural & social sciences study design

All studies must disclose on these points even when the disclosure is negative.

Study description	Quantitative prospective cohort study
Research sample	56468 participants of the Danish Diet, Cancer, and Health study, recruited between December 1993 to May 1997 from the greater area of Copenhagen and Aarhus, Denmark. Participants are both male and female (47.6% male) and between the ages of 50-65 years.
Sampling strategy	All participants of the Danish Diet, Cancer, and Health study cohort were included.
Data collection	All Danish residents are provided with a unique and permanent civil registration number enabling cross-linking between nationwide registers and the Danish Diet, Cancer, and Health cohort on the individual level. The following databases were cross-linked to the cohort: The Civil Registration System which includes data on age, sex, and vital status; The Integrated Database for Labor Market Research which contains information on annual income since 1980; The Danish Register of Causes of death with information on cause of death since 1994 by International Classification of Diseases (ICD) codes; and The Danish National Patient Register (DNPR) which holds information on all hospital admissions in Denmark since 1978. All diagnoses were defined by ICD using the 8th revision (ICD-8) until 1993 and the 10th revision (ICD-10) since 1994.
Timing	Participants were recruited between December 1993 to May 1997 and followed up for a maximum of 23 years, from the date of enrolment until the date of death, emigration, or end of follow-up (August, 2017), whichever came first.
Data exclusions	All exclusions were decided upon within the study protocol, a priori. Participants for whom information was missing or implausible (n=215) and those with extreme energy intakes [<2 092 kJ/day (<500 kcal/day) and >20 920 kJ/day (>5000 kcal/day)] (n=205), were excluded from the analysis. When investigating cardiovascular disease-related mortality we excluded participants with cardiovascular disease at baseline and when investigating cancer-related mortality we excluded participants with cancer at baseline.
Non-participation	There was no loss to follow-up.
Randomization	NA

Reporting for specific materials, systems and methods

We require information from authors about some types of materials, experimental systems and methods used in many studies. Here, indicate whether each material, system or method listed is relevant to your study. If you are not sure if a list item applies to your research, read the appropriate section before selecting a response.

Materials & experimental systems

n/a	Involved in the study	
☒	☐	Antibodies
☒	☐	Eukaryotic cell lines
☒	☐	Palaeontology
☒	☐	Animals and other organisms
☐	☒	Human research participants
☐	☒	Clinical data

Methods

n/a	Involved in the study	
☒	☐	ChIP-seq
☒	☐	Flow cytometry
☒	☐	MRI-based neuroimaging

Human research participants

Policy information about studies involving human research participants

Population characteristics	See above.
Recruitment	Participants residing within the greater areas of Copenhagen and Aarhus, identified as being free from cancer at baseline through a search in the cancer registry, were invited to participate in the Danish Diet Cancer and Health study. Participants in the study were likely to be of a higher socioeconomic status than non-participants. As participants with a low flavonoid intake were likely to be of a lower socioeconomic status, and the greatest difference in risk of mortality was seen between the lowest

	quintiles of flavonoid intake, our results would likely be strengthened had our cohort been more representative of the entire Danish population.
Ethics oversight	In Denmark, register studies do not require approval from ethics committees. The Danish Data Protection Agency approved this study (Ref no 2012-58-0004 I-Suite nr: 6357, VD-2018-117). Informed consent was obtained from all participants to search for information from medical registries.

Note that full information on the approval of the study protocol must also be provided in the manuscript.

Clinical data

Policy information about clinical studies

All manuscripts should comply with the ICMJE guidelines for publication of clinical research and a completed CONSORT checklist must be included with all submissions.

Clinical trial registration	NA
Study protocol	Tjønneland, A. et al. Study design, exposure variables, and socioeconomic determinants of participation in Diet, Cancer and Health: a population-based prospective cohort study of 57,053 men and women in Denmark. Scand J Public Health 35, 432-441 (2007).
Data collection	Information on sex, age, and lifestyle factors such as smoking, alcohol consumption, and daily activity were obtained using the self-administered questionnaire at study enrolment. Clinical measurements such as BMI and total cholesterol were taken at the study centers. Annual income was used as a proxy for socio-economic status and was defined as household income after taxation and interest, for the value of the Danish currency in 2015. Income, grouped in quartiles, was estimated as the mean income of 5 years up to and including the year of study inclusion. Self-reported myocardial infarction and self-reported stroke at baseline were combined with ICD codes of ischemic heart disease and ischemic stroke, respectively, dated prior to participant enrolment. ICD codes dated prior to participant enrolment were used to identify baseline comorbidities of peripheral artery disease, chronic kidney disease, chronic obstructive pulmonary disease, heart failure, atrial fibrillation, and cancers. For hypertension and diabetes mellitus self-reported prevalence was used. Prevalent CVD was defined by the presence of at least one diagnosis of ischemic heart disease, peripheral artery disease, ischemic stroke, heart failure, or atrial fibrillation prior to recruitment.
Outcomes	Vital status and date of death for every participant was obtained from the Civil Registration System. Cause of death data was obtained from the National Death Register. CVD-related mortality was defined as any ICD-10 diagnoses registered as a cause of death related to CVD (I00-I99) and cancer-related mortality was defined as any ICD-10 diagnosis registered as a cause of death related to cancer (C00-C99), dated after participant enrolment.
